# Shelf-Life of Half-Shell Mussel (*Mytilus edulis*) as Affected by Pullulan, Acidic Electrolyzed Water, and Stable Chlorine Dioxide Combined Ice-Glazing during Frozen Storage

**DOI:** 10.3390/foods10081896

**Published:** 2021-08-16

**Authors:** Yuanpei Gao, Huili Jiang, Dandan Lv, Soottawat Benjakul, Bin Zhang

**Affiliations:** 1 Key Laboratory of Health Risk Factors for Seafood of Zhejiang Province, College of Food Science and Pharmacy, Zhejiang Ocean University, No. 1, Haida South Road, Lincheng Changzhi Island, Zhoushan 316022, China; gaoyp89@zjou.edu.cn (Y.G.); jianghl1105@163.com (H.J.); dandanlv0012@163.com (D.L.); 2 International Center of Excellence in Seafood Science and Innovation, Faculty of Agro-Industry, Prince of Songkla University, Songkhla 90112, Thailand; soottawat.b@psu.ac.th

**Keywords:** acidic electrolyzed water (AEW), chlorine dioxide (ClO_2_), frozen storage, glazing, mussel (*Mytilus edulis*), pullulan

## Abstract

Mussel (*Mytilus edulis*) is an economic shellfish with a high nutritional value. Due to the high amount of protein and fat, fresh mussels are susceptible to spoilage during storage. In the present study, how a combination of pullulan, acidic electrolyzed water (AEW), and stable chlorine dioxide (ClO_2_) ice-glazing treatments affect the quality of mussels was investigated during 90 days of frozen storage. The results indicate that the combined glazing treatment effectively maintained the mussel muscle quality during storage mainly due to its air barrier actions. Mussel samples coated with AEW and ClO_2_ showed lower aerobic plate counts than other groups, resulting from the strong antibacterial action of AEW and ClO_2_. After 90 days of frozen storage, the mussel glazed with a combination of AEW, ClO_2_, and pullulan solutions showed better texture properties, higher content of myofibrillar proteins, higher Ca^2+^-ATPase activity, and more SH groups than the other glazing treatments. The water-holding capacity and SEM observations showed that the pullulan glazing efficiently inhibited the physical damage caused by the frozen and long-term storage, which mainly contributed to the high amount of hydrophilic hydroxyl groups in the muscle tissues. The present study supports the use of a combination of cryoprotectants for extending the shelf-life of frozen mussel products during long-term storage.

## 1. Introduction

Mussel, a kind of shellfish with important health and economic value, is only rich in protein, fat, and essential amino acids. In addition, it has a unique flavor, which is appreciated by consumers [1]. Mussels are easily cultivated on a large scale. Its vast aquaculture areas are distributed in most coastal provinces of China. However, due to the high protein content, mussels are easily spoiled by microorganisms during transportation and storage. Therefore, low-temperature treatment is often used to extend the storage period of mussels. Air frozen storage is the most widely used method in seafood because of its convenience and low energy consumption [2]. During frozen or cold storage, however, mussel products may experience surface drying and dehydration, which may cause freezer burn. Moreover, the quality may decline, due to oxidation or rancidity [3]. On the other hand, some psychrophilic microorganisms remain on the mussels during the freezing process. These microorganisms not only affect the edible safety of mussels but also affect their quality during storage.

Water ice-glazing is widely used in the frozen seafood industry and can be defined as applying a layer of ice on the surface of frozen products by spraying or brushing on water or immersing the product in a water bath. The layer covers the product, shielding it from oxidation of lipids and proteins and spoilage by microorganisms. Thus, the technique extends the shelf-life of frozen products. Pullulan is an exopolysaccharide widely used in the food industry, and it possesses potential applications, such as a glazing agent and for cryoprotectance [4]. It is a water-soluble polysaccharide and a biodegradable polymer with many applications in medicine and biotechnology. In recent years, it has been employed in food packaging, resulting from its proven film-forming ability [5] due to it being an edible polymer without any toxicity or carcinogenicity. Pullulan is not easily assimilated by bacteria, molds, and fungi. It can be mixed with a range of food or non-food materials to enhance the shelf-life of products which protects them from degradation [6]. Acidic electrolyzed water (AEW) and steady-state chlorine dioxide (ClO_2_) are internationally recognized as safe, non-toxic disinfectants [7,8]. Wang et al. [9] applied AEW to frozen shrimp and demonstrated that AEW inhibited alterations in pH, bacterial counts, diversity of bacterial flora, and muscle fiber contraction of shrimp. In addition, AEW served as a potential substance for decreasing the activity of cathepsin B and polyphenol oxidase. Lin et al. [10] also proved that AEW ice can mitigate changes in pH, color, and the formation of total volatile basic nitrogen of shrimp. Chung et al. [11] investigated how HClO and ClO_2_ affect the disinfection of fresh vegetables and fruits, and the authors suggested that ClO_2_ efficiently decreased the total bacterial counts, compared to the HClO treatment. Trinetta et al. [12] also demonstrated that ClO_2_ plays an important role in disinfection without residue after treatment.

In recent years, much attention has been paid to the preservation effect of glazing, pullulan, AEW, and ClO_2_ individually, and their ability to disinfect has been investigated. However, few studies focused on the combined effect of glazing and pullulan, AEW, and ClO_2_ on the preservation of frozen seafood. Therefore, the present study treated mussels with a combination of pullulan, AEW, and ClO_2_ glazing, and the effect on the mussel, with high protein content, during frozen storage was evaluated.

## 2. Material and Approaches

### 2.1. Chemicals, Microorganisms and Mussel Samples

Pullulan (C_20_H_36_O_16_, food grade, purity > 98%) and stable chlorine dioxide (ClO_2_, food grade, purity > 98%) came from Sigma-Aldrich (St. Louis, MO, USA) and Chemical Reagents Shanghai Co., Ltd. (Shanghai, China), respectively. Glutaraldehyde, paraformaldehyde, acetic acid, boric acid, alcohol, tris(hydroxymethyl)aminomethane (Tris), sodium chloride, perchloric acid, and formalin came from Seebio Biotech Co., Ltd. (Shanghai, China). Violet red bile agar (VRBA), baird-parker agar base (BPAB), and plate count agar (PCA) came from Shandong Tuopu Bio-engineering CO., Ltd. (Zhaoyuan, China).

Escherichia coli (ATCC 25922) and Staphylococcus aureus (ATCC 25923) strains (as the target representatives) were obtained from the Institute of Microbiology, Chinese Academy of Sciences (AS; Beijing, China). Every chemical employed in this research was analytical grade, except where otherwise specified.

Acidic electrolyzed water (AEW) was produced by electrolysis of 0.5% (*w*/*v*) NaCl using an electrolysis generator (FX-SWS100, Yantai Fangxin Water-Treatment Equipment Co., Ltd., China). In this experiment, strongly acidic (pH 4.5) and weakly acidic (pH 6.5) AEW solutions were prepared and used immediately for the subsequent glazing process.

Live blue mussel (*M. edulis*) specimens (each weighing 53–58 g and 10.8–11.5 cm in length, 3.8–4.1 cm in width, and 6.4–7.0 cm in height) were collected from a pristine site three miles off the coast in Zhoushan, Zhejiang province, China. A total of 60 mussels were procured in each treatment and randomly collected in each measurement. The mussel samples were packed into cold boxes (4 °C) and transported to the Zhejiang Ocean University within approximately 15–20 min from the moment of packaging. Upon arrival, the shells were cleaned thoroughly with tap water to remove the debris. Then, the mussels were drained for 1 min and steam shucked for 5 min. Finally, the mussels on half-shell (whole mussel meat connected with one half-shell) were obtained manually with a sterile knife.

### 2.2. Glazing Process and Frozen Storage

The half-shell mussels were maintained at −25 °C for 6 h in a refrigerator. Then, the frozen samples were randomly assigned to seven different groups, which were then immersed in the prepared glazing solutions. The glazing solutions included the control (frozen mussels without extra treatment), fresh water (as the negative control), fresh water containing 0.5% (*w*/*v*) pullulan, AEW (pH 4.5 and 6.5) containing 0.5% (*w*/*v*) pullulan, fresh water containing 0.015 g/L ClO_2_ and 0.5% (*w*/*v*) pullulan, and fresh water containing 0.030 g/L ClO_2_ and 0.5% (*w*/*v*) pullulan. The glazing treatment was performed according to the industrial process and CODEX STAN procedure under the following conditions: immersion time, 10 s; mussel temperature, −25 °C; and solution temperature, 1 °C. After glazing, the glazed mussel samples were immediately put into polystyrene trays, which were manually placed into polyethylene bags (150 μm thickness, 32 × 22 cm). Next, all packaged samples were stored in a −18 °C refrigerator for 90 days of storage. After the corresponding time, the frozen samples were taken out and thawed at 4 °C for 3 h in a refrigerator before analysis.

### 2.3. Microbiological Analysis

Prior to the immersion glazing, the prepared half-shell mussels were incubated in infection solutions (0 °C to 4 °C) for 10 min. These solutions contained 103 cfu/mL of Escherichia coli (*E. coli*) and 103 cfu/mL of Staphylococcus aureus (*S. aureus*). Next, the infected samples were removed and drained for 1 min, prior to freezing and glazing procedures. During frozen storage, the aerobic plate counts (cfu/g), S. aureus (cfu/g), and *E. coli* counts (cfu/g) of the glazed mussels were evaluated using the National Food Safety Standard Methods of China [13,14,15]. Briefly, after storage, 25 g of sample was homogenized with 225 mL stroke-physiological saline solution. The obtained homogenate was serially diluted before plating and incubated at 30 °C for 72 h before counting. Each dilution was determined in triplicates. The medium of culture was Mueller Hinton agar. Sterilized diluent was used as blank for the control test, and the results were recorded and expressed as CFU/g.

### 2.4. Texture Profile Analysis

The texture performances (encompassing springiness and chewiness) of muscle tissues were determined with a texture analyzer (TMS-Pro, Sterling City, VA, USA) based on the approach of Zhang et al. [16]. The device was equipped with a cylindrical steel probe (P/50) with a trigger force of 0.6 N. The mussel samples were compressed to 30% of the height at a constant speed of 1.0 mm/s for 3 s.

### 2.5. Weight Loss Analysis

The weight loss of glazed half-shell mussels induced by sublimation was measured by weighing the samples before and after frozen storage. First, the mussel samples were weighed accurately (defined as M0; 0.01 g) immediately after the glazing process (0 days). During storage intervals, the weight of the glazed mussels was calculated and specified as Mx (x = 15, 30, 45, 60, 75, or 90; 0.01 g). Weight loss was calculated as follows:

weight loss (%) = [(M0 − Mx)/M0] × 100(1)

### 2.6. Determination of Total Volatile Base Nitrogen (TVBN) Content

TVBN content in half-shell mussel muscle was determined using a steam-distillation of trichloroacetic acid (TCA)-mussel extract, according to the report by Zhang et al. [17]. The TVBN extract of mussel samples in 0.60 mol/L TCA solution was loaded into a steamed Kjeldahl-type distillation tube and then absorbed by 0.50 mol/L boric acid solution. The boric acid distillation was titrated with 0.02 mol/L HCl solution, and TVBN content was expressed as mg/100 g mussel muscle.

### 2.7. Determination of Myofibrillar Protein Content and Ca^2+^-ATPase Activity

According to the approach described by Zhang et al. [18], myofibrillar proteins extracted from the mussel muscle were prepared. Briefly, the mussel muscle was minced and homogenized in five volumes of 20 mmol/L Tris-maleate buffer (pH 7.0) containing 0.05 mol/L KCl employing a homogenizer for 1 min (4 °C). The mixture was centrifuged at 10,000× *g* for 10 min, and the sediment was resuspended in the same buffer, homogenized, and extracted once more. Finally, the resulting sediment was added to 10 volumes of the same buffer, homogenized, and centrifuged at 6000× *g* for another 10 min (4 °C). The obtained supernatant was recognized as the extracted myofibrillar protein (mg/g).

Based on a previously published procedure [19], myofibrillar Ca^2+^-ATPase activity was determined. The extracted myofibrillar proteins were combined with 0.50 mol/L Tris-maleate buffer (pH 7.0) containing 0.10 mol/L CaCl_2_ and 20 mmol/L adenosine 5′-triphosphate. The final concentrations of myofibrillar proteins in the buffer ranged from 1.0 to 2.0 mg/mL proteins. After incubation in a water bath (30 °C, 5 min), 30% (*w*/*v*) TCA solution was added to the mixture to terminate the reaction. The mixture was centrifuged at 5000× *g* for 5 min. Ca^2+^-ATPase activity of myofibrillar proteins was computed as the micromoles of inorganic phosphate released in the supernatant per milligram of protein per minute (µmol Pi/mg/mi).

### 2.8. Determination of Total Sulfhydryl Content

Based on the approach of Kong et al. [20], total sulfhydryl (SH) content was determined. Briefly, 0.5 mL extracted myofibrillar protein solution (4 mg/mL) was mixed with 4.5 mL of 0.2 mol/L of Tris-HCl buffer (containing 8 mol/L urea, 2% SDS, 10 mmol/L EDTA; pH = 8.0). Then, 4 mL of mixture was combined with 0.5 mL of 0.1% 5, 5′-dithio-bis (2-nitrobenzoic acid) in 0.2 mol/L of Tris-HCl buffer, and the mixture was incubated for 25 min at 40 °C. The absorbance was determined at 412 nm employing a spectrophotometer. The total SH concentration was calculated from absorbance employing the molar extinction of 13,500 M^−1^·cm^−1^ and expressed as mmol/10 g protein.

### 2.9. Scanning Electron Microscopy (SEM) Analysis

The micro-structure of the muscle tissues was viewed employing an SEM (TM3030Plus, Hitachi Ltd., Tokyo, Japan), as described by Zhang et al. [21]. The sample tissues were fixed with glutaraldehyde, formaldehyde, and osmium tetroxide (OsO4). Subsequently, the fixed tissues were rinsed and then dehydrated with ethanol. Finally, the vacuum freeze-dried muscle tissues were coated with gold powder and examined by a scanning electron microscope.

### 2.10. Data Analysis

The outcomes from the measurements were subjected to statistical analysis in SPSS package 13.0 for Windows (SPSS Inc., Chicago, IL, USA). Comparison of treatment averages was performed by Duncan’s test, and a *p*-value of below 0.05 implied the means differed markedly. The data represent the means ± SD of three replicates.

## 3. Outcomes and Discussions

### 3.1. Microbiological Analysis

**Figure 1:**
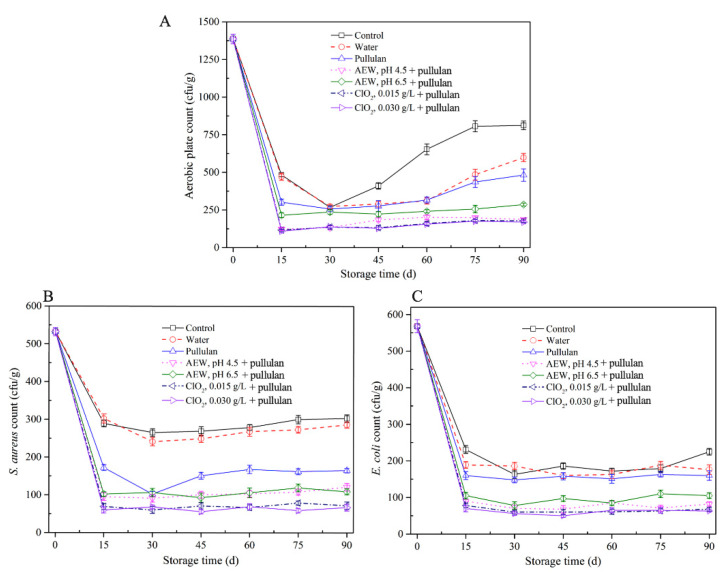
Microbiological properties of half-shell mussels treated with the control, water, and combined glazing solutions during 90 days of frozen storage. (**A**) aerobic plate counts; (**B**) *E. coli* counts; (**C**) *S. aureus* counts.

The results of microbiological qualities of half-shell mussel samples in the course of storage are exhibited (Figure 1). The aerobic plate count and *E. coli* and *S. aureus* counts in the control and water-glazed mussel groups decreased during the initial 0–15 days and then increased during storage. It is worth noting that the microorganism content in mussels with pullulan, AEW, and ClO_2_ treatment was lower than that with control and water treatment. After 30 days of storage, the aerobic plate counts in mussels showed no significant difference (*p* > 0.05) with or without glazing. The result is similar to that of He et al. [22] who claimed that water-glazing alone could not significantly decrease (*p* > 0.05) the total colony counts of sashimi samples during short-period storage. After 30 days, the aerobic plate counts in the control group increased faster than the glazing groups, due to the ice layer protecting the samples from environmental microbial contamination. The aerobic plate counts decreased to 310 cfu/g, 112 cfu/g, 236 cfu/g, 107 cfu/g, and 97 cfu/g after treatment with pullulan, AEW pH 4.5 + pullulan, AEW pH 6.5 + pullulan, ClO_2_ 0.0015 g/L + pullulan, and ClO_2_ 0.0030g/L + pullulan in the first 15 days storage, respectively. From Figure 1B,C), *E. coli* and *S. aureus* counts decreased in the first 15 days and remained stable as storage progressed. This is likely because the low temperature inhibited the growth of both bacterial species. Notably, the AEW and ClO_2_ treatments significantly decreased (*p* < 0.05) the *E. coli* counts, and pullulan treatment decreased the *S. aureus* counts during storage. From the results, the antibacterial ability of the combination of pullulan + glazing group was slightly better than that of the control and only glazing, and weaker than AEW and ClO_2_. Pullulan coating did not effectively kill microorganisms [23] but inhibited the growth of microorganisms after 90 days of storage. AEW and ClO_2_ have strong antibacterial abilities, and their abilities increased with decreasing pH and increasing concentration, respectively.

### 3.2. Texture Analysis

**Figure 2:**
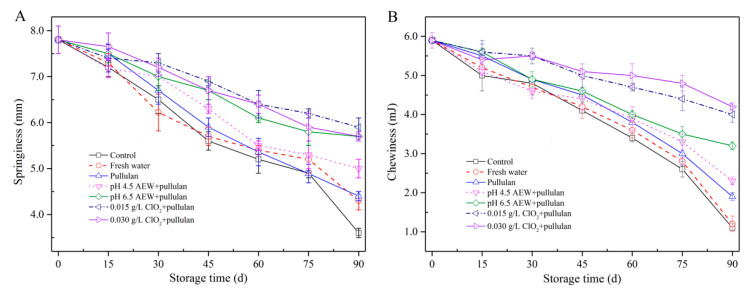
Texture properties of half-shell mussels treated with the control, water, and combined glazing solutions during 90 days of frozen storage. (**A**) springiness; (**B**) chewiness.

Texture is an important parameter to evaluate the quality of food products. The springiness and chewiness of mussels during storage are shown in Figure 2. In the present study, the springiness (Figure 2A) of mussels with treatments of AEW (pH 6.5) + pullulan and ClO_2_ (0.015 g/L, 0.030 g/L) + pullulan was significantly higher (*p* < 0.05) than in other groups after 90 days of storage, followed by AEW (pH 4.5) + pullulan, pullulan, water, and control group. The chewiness (Figure 2B) showed a similar trend as the springiness. From the results, both springiness and chewiness gradually decreased with increasing storage time. Frozen storage results in protein denaturation of seafood. From a previous study, the changes in protein structure decrease the interaction of protein and protein or protein and water in the seafood. However, the formation of ice crystals physically destroys the cell structure during frozen storage and results in the release of cytoplasm after thawing. Consequently, frozen storage decreases the product texture [21]. During storage, the best texture of mussel muscle was observed in the group treated with ClO_2_ + pullulan, followed by AEW + pullulan, pullulan, water, and the control. Microorganisms could decompose the proteins that maintain the texture of food. In the present study, the combination of glazing and antibacterial solution efficiently inhibited the growth of bacteria to maintain the texture of mussels.

### 3.3. Weight Loss Analysis

**Figure 3:**
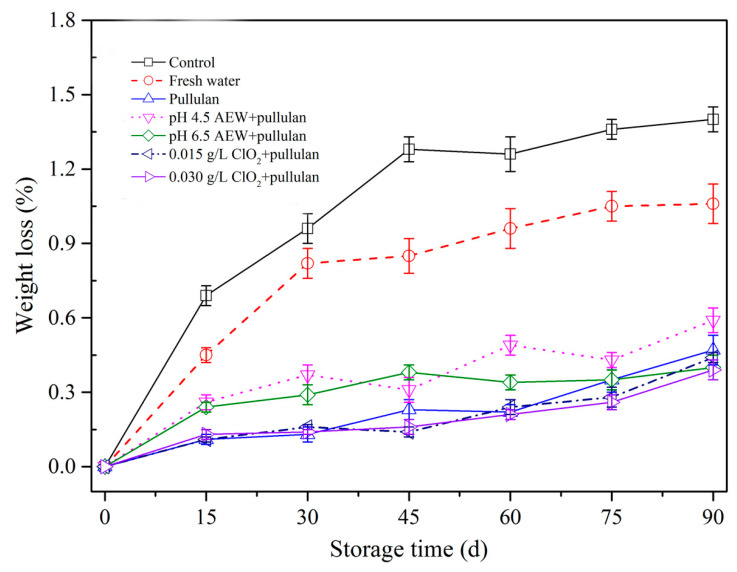
Weight loss of half-shell mussels treated with the control, water, and combined glazing solutions during 90 days of frozen storage.

Frozen storage results in weight loss of meat products because of moisture sublimation. Figure 3 shows the weight loss of mussels with different treatments. From the figure, greater weight loss was observed with increasing storage duration. This outcome aligns with Solval et al. [24] that demonstrated weight loss of frozen shrimp rose as frozen storage period rose. During frozen storage, the formation and growth of ice crystals resulted in the suppression, aggregation, and denaturation of protein. Consequently, the bound water was converted to free water. However, ice crystals destroy the cell membrane structure and increase the weight loss after thawing. After 90 days of storage, weight loss of mussels without any treatments was significantly higher (*p* < 0.05) than the other treatment groups, followed by water and AEW (pH 4.5). In addition, less weight loss was observed for the pullulan, AEW (pH 6.5) + pullulan, ClO_2_ (0.015 g/L) + pullulan, and ClO_2_ (0.030 g/L) + pullulan treatment groups. The weight loss of frozen aquatic products results from the denaturation of proteins and recrystallization of ice crystals in the process of frozen storage, causing sublimation of water. Glazing solutions form a layer on the surface of the mussel to prevent protein denaturation (see Section 3.5 and Section 3.6) and mussel dehydration. Therefore, the mussels with different glazing solutions effectively maintained weight during frozen storage. Interestingly, pullulan treatment has been reported to protect frozen food from sublimation due to its viscosity [4]. Consequently, it can be used to minimize weight loss of mussels during frozen storage.

### 3.4. pH and TVBN Content Analysis

**Figure 4:**
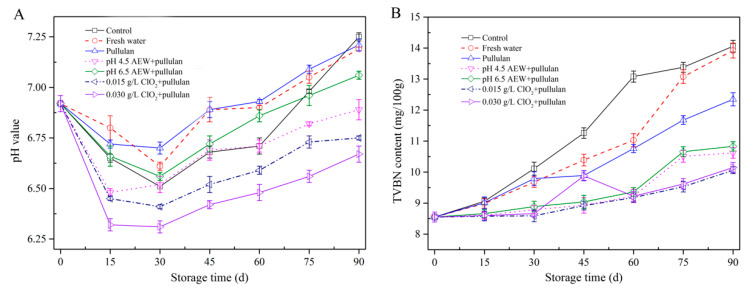
pH value (**A**) and TVBN content (**B**) of half-shell mussels treated with the control, water, and combined glazing solutions during 90 days of frozen storage.

The pH value can be used to determine the freshness of mussels. Figure 4A shows the changes in pH of mussels during frozen storage. The pH value of all groups dropped in the first 30 days of storage and increased gradually as storage time increased. After the death of mussels, the carbohydrate in the mussels is degraded into acidic substances, resulting in a decrease in pH. The pH value increased after 30 days storage because of the presence of amine compounds, which are produced by the oxidation of muscle protein or lipid [25]. After 90 days of storage, the pH of mussel with ClO_2_ + pullulan treatment was significantly lower (*p* < 0.05) than the other groups, followed by that of the AEW treatment and pullulan treatment groups. This could be attributed to the antibacterial activity of CIO2, AEW, and pullulan to prevent protein decomposition from microorganisms during storage.

Figure 4B exhibits the alterations in TVBN of mussels during storage. TVNB has been employed as a key parameter of seafood quality as it is directly associated with spoilage by bacteria. The TVBN value of mussel for all treatment groups rose as storage time rose; however, the TVBN of mussels in the control and water glazing group were significantly faster (*p* < 0.05) than other groups. Similar to the pH value, the TVBN of mussel with ClO_2_ + pullulan treatment was significantly lower (*p* < 0.05) than other groups, followed by that with AEW + pullulan treatment and pullulan treatment after 90 days storage. The glazing treatment isolated the mussels from the external environment, which slowed the oxidative deamination reaction, thereby reducing the amount of TVBN. In addition, both ClO_2_ + pullulan and AEW + pullulan treatments have excellent antibacterial abilities [11], which can effectively suppress the bacteria growth and lessen the decomposition of mussel protein. Consequently, these treatments decreased the pH and TVBN value of mussels.

### 3.5. Myofibrillar Protein Concentration and Ca^2+^-ATPase Activity Analysis

**Figure 5:**
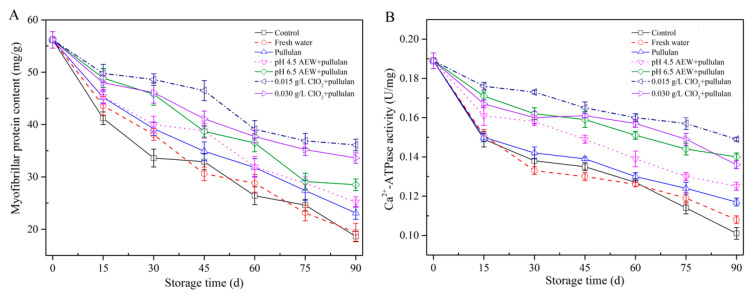
Myofibrillar protein content (**A**) and Ca^2+^-ATPase activity (**B**) of half-shell mussels treated with the control, water, and combined glazing solutions during 90 days of frozen storage.

Myofibrillar proteins, the major component of fish protein, are prone to denaturation in frozen storage, which changes the structure and functional characteristics of protein, causing the deterioration of fish quality. The myofibrillar protein content of mussels during storage is shown in Figure 5A. From the figure, the initial protein content was 56.5 mg/g. After 90 days of storage, the lowest myofibrillar protein contents of mussels were observed in the control and the water glazing group, followed by the pullulan, AEW (pH 4.5) + pullulan, AEW (pH 6.5) + pullulan, ClO_2_ (0.03 g/L) + pullulan, and ClO_2_ (0.015 g/L) + pullulan. A decrease in myofibrillar protein concentration caused the deterioration of texture and a reduction in the water holding capacity [26]. The samples treated with pullulan, AEW + pullulan, and ClO_2_ + pullulan groups had significantly decreased (*p* < 0.05) myofibrillar protein content, compared to other groups during storage. This could be related to the formation of ice crystals, irreversibly destructing the myofibrils. Ca^2+^-ATPase activity of myofibrillar protein showed a similar tendency as the myofibrillar protein content (Figure 5B). Ca^2+^-ATPase activity serves as an indicator of frozen denaturation of myofibrillar protein, and denaturation of proteins may occur when hydrophobic interaction, hydration of polar residues, and hydrogen bonds are destroyed. Jiang and Wu [27] studied the freezing-resistance of pullulan treatment in grass carp and demonstrated that pullulan addition curbed the denaturation of grass carp myofibrillar protein in frozen storage.

### 3.6. Total Sulfhydryl Concentration Analysis

**Figure 6:**
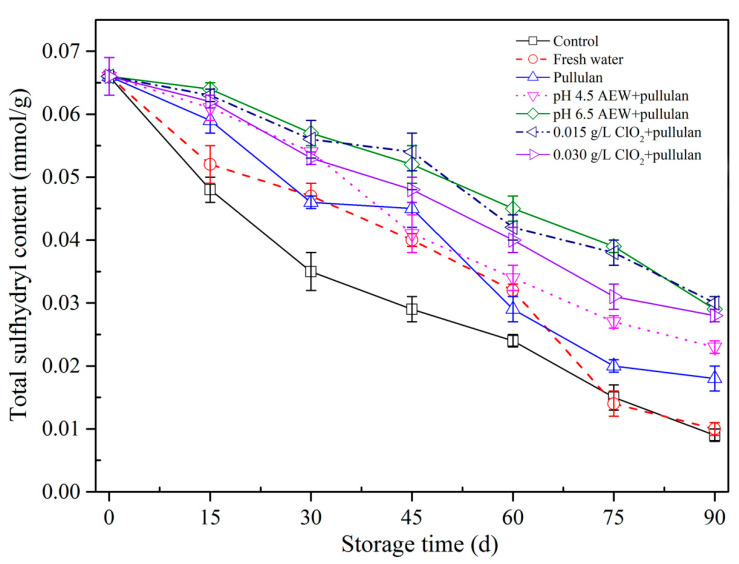
Total sulfhydryl content of half-shell mussels treated with the control, water, and combined glazing solutions during 90 days of frozen storage.

The alterations in sulfhydryl concentration of mussels during storage are exhibited (Figure 6). Sulfhydryl concentration of mussels with all treatments decreased. The total SH of every mussel dropped gradually as frozen storage time prolonged. The alterations in the spatial structure of myofibrillar protein resulting from ice crystal shaping caused the exposure of thiol groups at the intramolecular level, causing oxidation of thiol groups by the formation of disulfide bonds and the drop in sulfhydryl concentration in frozen storage [28,29]. Before 60 days of storage, the SH concentration in the control group was significantly below (*p* < 0.05) the other glazing groups, which suggested that glazing of mussel can slow SH concentration alterations and prevent the protein from oxidation compared with the unglazed mussels. At the end of storage, the mussels treated with AEW (pH 6.5) + pullulan, ClO_2_ (0.015 g/L) + pullulan, and ClO_2_ (0.030 g/L) + pullulan had the highest content of SH after storage. This could be because the ClO_2_ and AEW treatments have reducing capabilities, meaning they combine with free radicals and protect proteins from oxidation. The results show that glazing could prevent the mussel from oxidation, and ClO_2_ and AEW exhibited an obvious cryoprotective impact on myofibrillar protein of mussels in frozen storage at −18°C.

### 3.7. SEM Analysis

**Figure 7:**
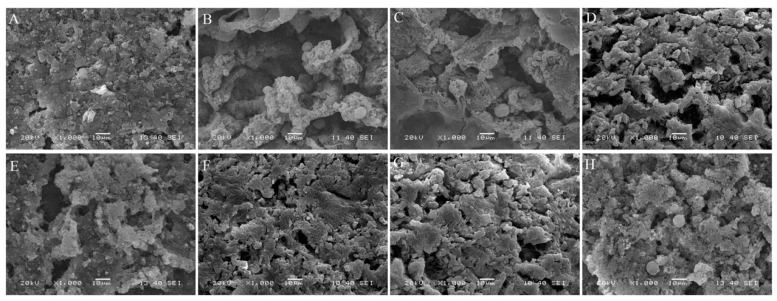
SEM images of mussel muscle tissues treated with the control (**B**), water (**C**) and combined glazing solutions after 90 days of frozen storage, compared with the fresh muscle tissue ((**A**), 0 days). (**D**), 0.5% (*w*/*v*) pullulan; (**E**), AEW pH 4.5; (**F**), AEW pH 6.5; (**G**), 0.015 g/L ClO_2_ and 0.5% (*w*/*v*) pullulan; and (**H**), 0.030 g/L ClO_2_ and 0.5% (*w*/*v*) pullulan.

The microscopic observation of mussels before and after frozen storage is shown in Figure 7. From Figure 7A, the fresh mussel muscle had relatively complete muscle structure without strips or breaks. After 90 days of frozen storage, mussel muscle in all groups was damaged. The changes in the muscle structure of meat products during the freezing process mainly result from shaping ice crystals. The growth of ice crystals in the freezing process destroys the cell structure, resulting in the deterioration of muscle quality after thawing [30]. The mussel muscle of the control group showed the largest gaps after frozen storage, followed by the pullulan (D), AEW pH 4.5 + pullulan (E), AEW pH 6.5 + pullulan (F), 0.015 g/L ClO_2_ + pullulan and 0.5% pullulan (G), and 0.030 g/L ClO_2_ and 0.5% pullulan (H). Kan et al. [31] found that hydrophilic hydroxyl groups in trehalose could combine with moisture molecules in muscle and protect the microstructure of muscle from frozen storage. In the present study, the addition of pullulan increased the stability of the glazing phase, due to its viscosity. Conversely, pure water glazing effectively reduced the moisture sublimination to avoid the formation of ice crystals (Figure 7G,H).

## 4. Conclusions

The present study focused on the quality changes of mussel muscle treated by different cryoprotectants during frozen storage. The results show that AEW and ClO_2_ could efficiently inhibit the growth of microorganisms in the mussel and protect the protein from oxidation during storage. Pullulan treatment improved the stability of the glazing phase due to its viscosity. The combined effect of cryoprotectants on the quality changes of mussels was clarified in this study, and the results show the use of a combination of cryoprotectants could extend the shelf-life of mussels.

## Data Availability

The datasets generated for this study are available on request to the corresponding author.

## References

[1] Grienke U., Silke J., Tasdemir D. (2014). Bioactive compounds from marine mussels and their effects on human health. Food Chem..

[2] Zhou X., Zhou D., Liu Z., Yin F., Liu Z., Li D., Shahidi F. (2019). Hydrolysis and oxidation of lipids in mussel Mytilus edulis during cold storage. Food Chem..

[3] Vanhaecke L., Verbeke W., Brabander H. (2010). Glazing of frozen fish: Analytical and economic challenges. Anal. Chim. Acta.

[4] Singh R.S., Kaur N., Kennedy J.F. (2019). Pullulan production from agro-industrial waste and its applications in food industry: A review. Carbohydr. Polym..

[5] Farris S., Unalan I.U., Introzzi L., Fuentes Alventosa J.M., Cozzolino C.A. (2014). Pullulan-based films and coatings for food packaging: Present applications, emerging opportunities, and future challenges. J. Appl. Polym. Sci..

[6] Singh R., Kaur N., Kennedy J. (2015). Pullulan and pullulan derivatives as promising biomolecules for drug and gene targeting. Carbohydr. Polym..

[7] Budzinska K., Dziedziak K., Szejniuk B., Traczykowski A., Michalska M., Berlec K., Pasela R. (2016). Effect of chlorine dioxide and hydrogen peroxide on elimination of Salmonella Enteritidis in wastewater from fruit and vegetable-processing industry. Przem. Chem..

[8] Phuvasate S., Su Y. (2010). Effects of electrolyzed oxidizing water and ice treatments on reducing histamine-producing bacteria on fish skin and food contact surface. Food Control..

[9] Wang M., Wang J., Sun X., Pan Y., Zhao Y. (2015). Preliminary mechanism of acidic electrolyzed water ice on improving the quality and safety of shrimp. Food Chem..

[10] Lin T., Wang J.J., Li J.B., Liao C., Pan Y.J., Zhao Y. (2013). Use of acidic electrolyzed water ice for preserving the quality of shrimp. J. Agric. Food Chem..

[11] Chung C.C., Huang T.C., Yu C.H., Shen F.Y., Chen H.H. (2011). Bactericidal effects of fresh-cut vegetables and fruits after subsequent washing with chlorine dioxide. Int. Proc. Chem. Biol. Environ. Eng..

[12] Trinetta V., Vaidya N., Linton R., Morgan M. (2011). Evaluation of chlorine dioxide gas residues on selected food produce. J. Food Sci..

[13] GB-4789.2 (2016). Aerobic Plate Count Examination.

[14] GB-4789.10 (2016). Food Microbiological Examination: Staphylococcus Aureus.

[15] NY/T-555 (2012). Detection Methods for Coliform Group, Faecal Coliform Group, Coliform Bacteria in Animal Products.

[16] Zhang B., Yang H., Tang H., Hao G., Zhang Y., Deng S. (2017). Insights into cryoprotective roles of carrageenan oligosaccharides in peeled whiteleg shrimp (*Litopenaeus vannamei*) during frozen storage. J. Agric. Food Chem..

[17] Zhang B., Ma L., Deng S., Xie C., Qiu X. (2015). Shelf-life of pacific white shrimp (*Litopenaeus vannamei*) as affected by weakly acidic electrolyzed water ice-glazing and modified atmosphere packaging. Food Control.

[18] Zhang B., Fang C., Hao G., Zhang Y. (2018). Effect of kappa-carrageenan oligosaccharides on myofibrillar protein oxidation in peeled shrimp (*Litopenaeus vannamei*) during long-term frozen storage. Food Chem..

[19] Zhang B., Zhao J., Chen S., Zhang X., Wei W. (2019). Influence of trehalose and alginate oligosaccharides on ice crystal growth and recrystallization in whiteleg shrimp (*Litopenaeus vannamei*) during frozen storage with temperature fluctuations. Int. J. Refrig..

[20] Kong C., Wang H., Li D., Zhang Y., Pan J., Zhu B., Luo Y. (2016). Quality changes and predictive models of radial basis function neural networks for brined common carp (*Cyprinus carpio*) fillets during frozen storage. Food Chem..

[21] Zhang B., Cao H., Wei W., Ying X. (2020). Influence of temperature fluctuations on growth and recrystallization of ice crystals in frozen peeled shrimp (*Litopenaeus vannamei*) pre-soaked with carrageenan oligosaccharide and xylooligosaccharide. Food Chem..

[22] He Q., Li Z., Yang Z., Zhang Y., Liu J. (2020). A superchilling storage–ice glazing (SS-IG) of Atlantic salmon (Salmo salar) sashimi fillets using coating protective layers of Zanthoxylum essential oils (EOs). Aquaculture.

[23] Bierhals V.S., Chiumarelli M., Hubinger M.D. (2011). Effect of cassava starch coating on quality and shelf life of fresh-cut pineapple (*Ananas comosus* L. Merril cv “Pérola”). J. Food Sci..

[24] Solval K.M., Rodezno L.A.E., Moncada M., Bankston J.D., Sathivel S. (2014). Evaluation of chitosan nanoparticles as a glazing material for cryogenically frozen shrimp. LWT Food Sci. Technol..

[25] Hu J., Xu Y., Majura J., Qiu Y., Ding J., Hatab S., Miao W., Gao Y. (2021). Combined Effect of the Essential Oil and Collagen Film on the Quality of Pacific Mackerel (*Pneumatophorus japonicus*) Fillet During Cold Storage. Foodborne Pathog. Dis..

[26] Zhang B., Hao G., Cao H., Tang H., Zhang Y., Deng S. (2017). The cryoprotectant effect of xylooligosaccharides on denaturation of peeled shrimp (*Litopenaeus vannamei*) protein during frozen storage. Food Hydrocoll..

[27] Jiang L., Wu S. (2018). Pullulan suppresses the denaturation of myofibrillar protein of grass carp (*Ctenopharyngodon idella*) during frozen storage. Int. J. Biol. Macromol..

[28] Sompongse W., Itoh Y., Obatake A. (1996). Effect of cryoprotectants and a reducing reagent on the stability of actomyosin during ice storage. Fish. Sci..

[29] Benjakul S., Visessanguan W., Thongkaew C., Tanaka M. (2003). Comparative study on physicochemical changes of muscle proteins from some tropical fish during frozen storage. Food Res. Int..

[30] Leygonie C., Britz T.J., Hoffman L.C. (2012). Impact of freezing and thawing on the quality of meat: Review. Meat Sci..

[31] Kan Z., Yan X., Ma J. (2015). Conformation Dynamics and Polarization Effect of α, α-Trehalose in a Vacuum and in Aqueous and Salt Solutions. J. Phys. Chem. A.

